# Ultrasound-guided, nursing-led, phase-based swallowing rehabilitation in elderly patients with MIBG-positive medullary thyroid carcinoma following ^131^I-MIBG therapy

**DOI:** 10.3389/fmed.2026.1775946

**Published:** 2026-06-22

**Authors:** Chunyan Hu

**Affiliations:** Center of Gerontology and Geriatrics, West China Hospital, Sichuan University/West China School of Nursing, Sichuan University, Chengdu, China

**Keywords:** aged, deglutition disorders, dysphagia rehabilitation, medullary thyroid carcinoma, nursing care, quality of life, radiopharmaceutical therapy adverse effects, ultrasonography

## Abstract

**Background:**

Medullary thyroid carcinoma (MTC) is a rare neuroendocrine malignancy originating from parafollicular C cells and often requires multimodal management, including surgery, targeted therapy, and radionuclide treatment. In elderly patients with MIBG-positive disease, ^131^I-MIBG therapy remains clinically valuable but is frequently associated with mucosal injury and neuromuscular dysfunction, resulting in dysphagia and malnutrition. Conventional swallowing rehabilitation often provides limited benefit in this population, underscoring the need for individualized, technology-assisted approaches. This study evaluated the association between ultrasound-guided, nursing-led, phase-based swallowing rehabilitation and clinical outcomes in elderly patients with MIBG-positive MTC following ^131^I-MIBG therapy.

**Materials and methods:**

This retrospective observational study analyzed 80 patients aged ≥65 years with post-therapy dysphagia (EAT-10 ≥ 3). Based on routine clinical practice, patients were categorized into a conventional rehabilitation group (*n* = 40) and an ultrasound-guided rehabilitation group (*n* = 40). Rehabilitation data over an eight-week period were analyzed. Outcomes included the Functional Oral Intake Scale (FOIS), MD Anderson Dysphagia Inventory (MDADI), EAT-10, ultrasound-derived suprahyoid muscle thickness and hyoid displacement, nutritional indices, aspiration events, and pneumonia-related readmissions.

**Results:**

The ultrasound-guided rehabilitation group demonstrated significantly greater improvements in FOIS (+2.1 ± 0.5 vs. + 1.2 ± 0.6; *p* < 0.001), MDADI (+32% vs. + 18%; *p* < 0.001), and EAT-10 (−6.8 ± 2.1 vs. − 3.4 ± 1.9; *p* < 0.001). Ultrasound parameters showed marked increases in suprahyoid muscle thickness (+24%) and hyoid displacement (+19%). Compared with the conventional rehabilitation group, aspiration events decreased from 15 to 9%, and pneumonia-related readmissions declined from 15 to 5%. No rehabilitation-related adverse events were observed.

**Conclusion:**

Participation in ultrasound-guided, nursing-led, phase-based swallowing rehabilitation was associated with significant improvements in swallowing function, muscle performance, nutritional status, and quality of life in elderly patients with MIBG-positive MTC after ^131^I-MIBG therapy. This approach appears safe, feasible, and scalable for geriatric oncology rehabilitation.

## Introduction

Medullary thyroid carcinoma (MTC) is a rare thyroid malignancy derived from parafollicular C cells and represents approximately 1–2% of all thyroid cancers (1, 2). Unlike differentiated thyroid carcinomas, MTC does not concentrate radioactive iodine, limiting therapeutic options in advanced or recurrent disease. For patients with MIBG-positive tumors, ^131^I-MIBG therapy remains an important targeted radionuclide treatment, particularly in elderly individuals who may not tolerate aggressive systemic therapies (3, 4). Despite its therapeutic value, ^131^I-MIBG therapy is frequently associated with adverse effects, including mucosal inflammation, salivary gland dysfunction, neuromuscular impairment, and radiation-induced fibrosis of the upper aerodigestive tract (5). Dysphagia is a common and debilitating consequence of these changes, especially in elderly patients who often have reduced physiological reserve and comorbid conditions. Dysphagia in this population is associated with malnutrition, aspiration pneumonia, prolonged hospitalization, and diminished quality of life (6). Conventional swallowing rehabilitation typically relies on standardized exercises and subjective clinical assessment. However, in elderly oncology patients, outcomes are often suboptimal due to limited adaptability of protocols and lack of objective, real-time feedback (7). The biomechanics of swallowing involve complex coordination of oral, pharyngeal, and esophageal phases, which can be profoundly disrupted by radiation-related neuromuscular injury (8). Point-of-care ultrasound (POCUS) has emerged as a non-invasive, bedside tool capable of visualizing swallowing-related structures, including tongue motion, suprahyoid muscle contraction, and hyoid bone excursion (9). Ultrasound enables dynamic, real-time assessment without radiation exposure and can be repeatedly applied in frail or immunocompromised patients. When integrated into rehabilitation programs delivered by trained nursing staff, ultrasound may enhance precision, individualization, and patient engagement (10).

Nursing-led rehabilitation models are increasingly recognized as effective and scalable strategies in geriatric and oncology care, particularly in settings with limited access to specialized speech-language pathology services (11). However, evidence supporting ultrasound-guided swallowing rehabilitation in thyroid cancer–related dysphagia remains sparse, with most prior studies focusing on head and neck cancers treated with external beam radiotherapy (12). The present study retrospectively evaluated the effectiveness, safety, and physiological correlates of ultrasound-guided, nursing-led, phase-based swallowing rehabilitation in elderly patients with MIBG-positive MTC following ^131^I-MIBG therapy.

## Materials and methods

### Ethics

This study was a retrospective observational analysis of existing clinical data. The study protocol was reviewed and approved by the Institutional Ethics Committee of West China Hospital of Sichuan University, Chengdu, Sichuan Province, China (Approval No. 2023-00YT-8). Owing to the retrospective design and use of anonymized data, the requirement for written informed consent was waived by the Ethics Committee. The study was conducted in accordance with the Declaration of Helsinki (2013 revision).

### Study design

This was a single-center, retrospective observational cohort study evaluating the association between ultrasound-guided, nursing-led, phase-based swallowing rehabilitation and clinical outcomes in elderly patients with MIBG-positive medullary thyroid carcinoma following ^131^I-MIBG therapy. Clinical, rehabilitation, and imaging data were extracted from electronic medical records and standardized nursing rehabilitation logs. The study was reported in accordance with the STROBE (Strengthening the Reporting of Observational Studies in Epidemiology) guidelines.

### Sample size consideration

As this was a retrospective observational study, no *a priori* sample size calculation was performed. The sample size was determined by the number of eligible patients with complete clinical and rehabilitation records available during the study period.

### Participants

Patients aged ≥65 years with a confirmed diagnosis of MIBG-positive medullary thyroid carcinoma who had completed ^131^I-MIBG therapy and subsequently developed dysphagia were eligible for inclusion. Dysphagia was defined as an Eating Assessment Tool-10 (EAT-10) score ≥3. Exclusion criteria included severe cognitive impairment precluding participation in rehabilitation, uncontrolled systemic comorbidities (such as advanced cardiac failure or poorly controlled diabetes), structural abnormalities unrelated to radionuclide therapy that could affect swallowing, and incomplete clinical or rehabilitation records.

Between May 5, 2023 and June 9, 2025, 80 eligible patients were retrospectively identified. All patients had completed their radionuclide therapy regimen and were receiving standard post-treatment medical and nutritional support at the time of rehabilitation.

### Rehabilitation exposure

**Figure 1:**
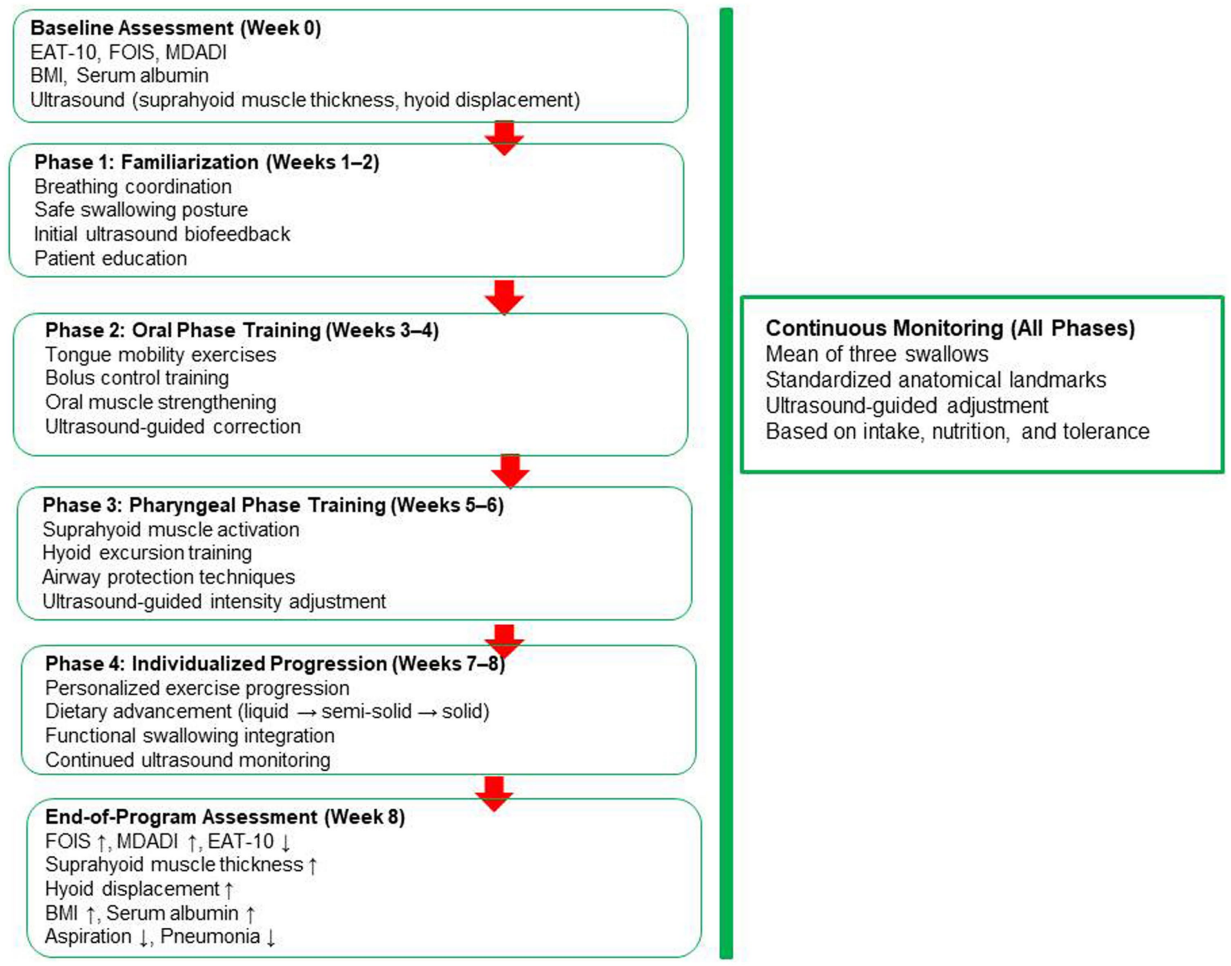
Phase-based, ultrasound-guided swallowing rehabilitation protocol. The 8-week program included four phases: familiarization (weeks 1–2), oral phase training (weeks 3–4), pharyngeal phase training (weeks 5–6), and individualized progression (weeks 7–8). Exercises were continuously adjusted using real-time ultrasound biofeedback and standardized monitoring protocols. Outcomes were assessed at week 8.

Based on routine clinical practice during the study period, patients were categorized into two groups: Conventional rehabilitation group (*n* = 40): Patients received standard swallowing rehabilitation consisting of conventional oropharyngeal exercises, respiratory coordination training, and postural strategies delivered by trained rehabilitation personnel. Ultrasound-guided rehabilitation group (*n* = 40): Patients participated in a structured, nursing-led, phase-based swallowing rehabilitation program incorporating real-time ultrasound guidance. The program targeted the oral, pharyngeal, and esophageal phases of swallowing. High-frequency linear ultrasound transducers (7–12 MHz) were used to visualize suprahyoid muscle contraction, tongue motion, and hyoid bone excursion, allowing individualized adjustment of exercise intensity and progression. Ultrasound assessments were performed with patients in a standardized seated position using predefined anatomical landmarks for submental and hyoid visualization. Each rehabilitation session lasted approximately 30–40 min and was conducted five times weekly over the eight-week rehabilitation period. Ultrasound-guided exercises were individualized according to swallowing performance, suprahyoid muscle contraction, and hyoid excursion observed during dynamic assessment. Measurements were obtained from the mean of three swallowing attempts to improve consistency and reduce measurement variability. Rehabilitation data over an eight-week treatment period were analyzed retrospectively. Because of the retrospective observational design, rehabilitation exposure was not assigned by investigators. Allocation to conventional or ultrasound-guided rehabilitation reflected routine clinical practice, rehabilitation resource availability, and clinician judgment during the study period. The ultrasound-guided rehabilitation program was progressively implemented within the institution during the later phase of the study period, and eligible patients were managed according to the rehabilitation approach available at the time of treatment. To reduce potential selection bias and baseline imbalance associated with this non-randomized design, multivariable adjustment and propensity-score weighting analyses were performed. Nursing staff supervising the ultrasound-guided rehabilitation program underwent institutional training in swallowing rehabilitation and point-of-care ultrasound (POCUS), including standardized instruction in image acquisition, anatomical landmark identification, swallowing biomechanics assessment, and rehabilitation adjustment protocols prior to participation in the program. All rehabilitation procedures were delivered by trained nursing staff as part of routine clinical care; data extraction and statistical analyses were performed by the author. The phase-based rehabilitation protocol is illustrated in Figure 1. Continuous monitoring and adjustment were performed across all phases based on ultrasound findings, functional intake, nutritional status, and patient tolerance.

### Outcome measures

The primary outcome was change in the Functional Oral Intake Scale (FOIS) score from baseline to the end of the eight-week rehabilitation period. Secondary outcomes included changes in MD Anderson Dysphagia Inventory (MDADI) scores, EAT-10 scores, ultrasound-derived measures of suprahyoid muscle thickness and hyoid displacement, nutritional indices (body mass index and serum albumin), aspiration events, and pneumonia-related hospital readmissions (13). Ultrasound measurements were recorded in standardized nursing logs using predefined anatomical landmarks. For each assessment, the mean of three swallows was used for analysis. Measurements were performed by nurses trained in point-of-care ultrasound (POCUS) under institutional protocols. To reduce detection bias, outcome data extraction and statistical analysis were performed using anonymized records. However, because ultrasound assessment was integrated into routine rehabilitation delivery, complete blinding of sonographers to rehabilitation exposure could not be guaranteed. This issue was therefore acknowledged as a study limitation.

### Statistical analysis

Statistical analyses were performed using SPSS version 26.0 (IBM Corp., Armonk, NY, USA) and R version 4.3.2. Continuous variables were expressed as mean ± standard deviation, and categorical variables as frequencies and percentages. Baseline group differences were initially evaluated using independent-samples t tests, chi-square tests, or Fisher’s exact tests as appropriate. Given the retrospective, non-randomized design, multivariable adjustment and propensity-score weighting were applied to minimize confounding bias. Covariates included in adjusted models were selected *a priori* based on clinical relevance and included age, sex, tumor stage, baseline body mass index, serum albumin, baseline FOIS score, baseline EAT-10 score, and baseline MDADI score. Analysis of covariance (ANCOVA), linear mixed-effects models, and logistic regression were used as appropriate. Changes in continuous outcomes were analyzed using ANCOVA and linear mixed-effects models with rehabilitation group as the fixed effect and baseline values entered as covariates. Logistic regression models were used for binary clinical outcomes, including aspiration events and pneumonia-related readmissions. Propensity scores were estimated using multivariable logistic regression, and stabilized inverse probability of treatment weighting (IPTW) was applied. Covariate balance before and after weighting was assessed using standardized mean differences (SMDs). Standardized mean differences (SMDs) < 0.10 were considered indicative of adequate balance. Sensitivity analyses using weighted and unweighted models demonstrated consistent effect estimates across major clinical outcomes. Owing to the relatively small number of aspiration and pneumonia events, odds ratios and associated confidence intervals were interpreted cautiously. A two-sided *p* value <0.05 was considered statistically significant.

## Results

### Patient identification and baseline characteristics

**Figure 2:**
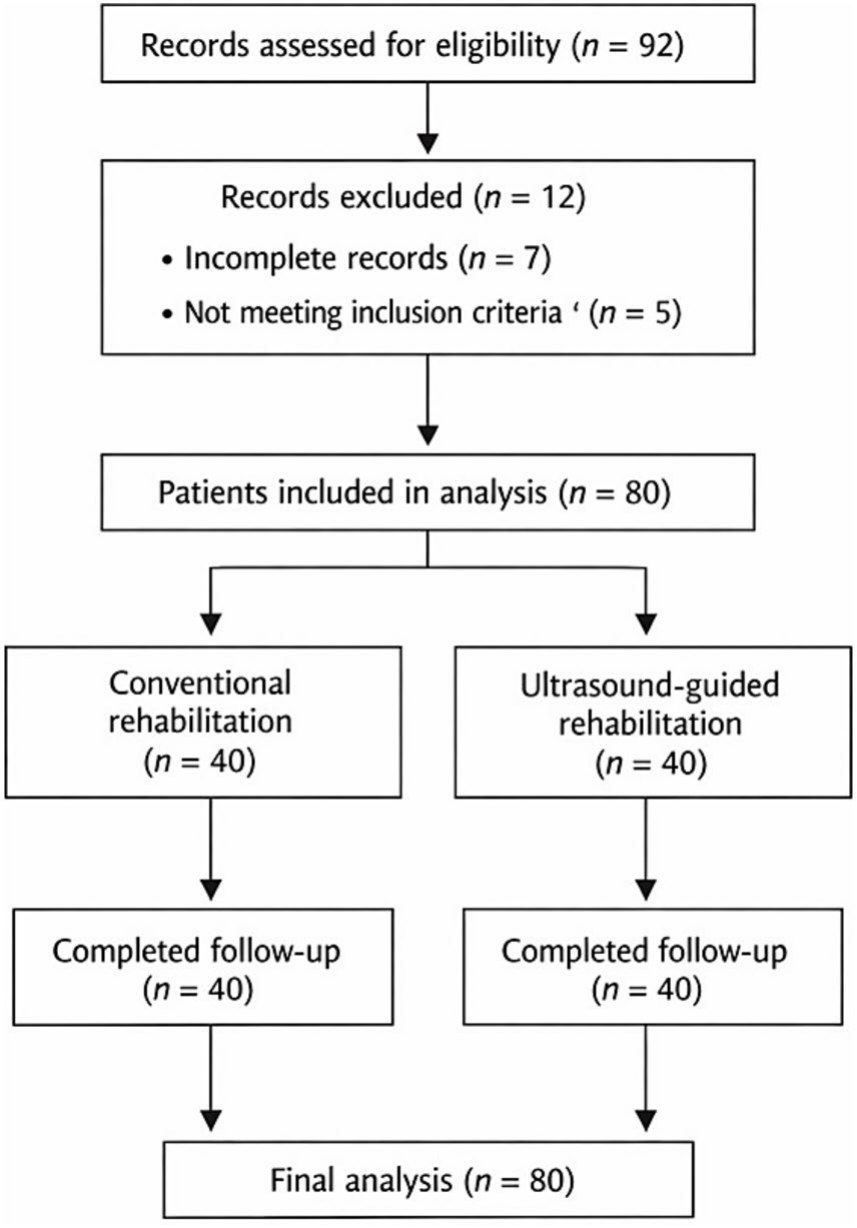
Flow diagram of patient screening, inclusion, group classification, and analysis. A total of 92 patients were screened; 12 were excluded due to incomplete records or eligibility criteria. Eighty patients were retrospectively identified and categorized according to routine clinical practice into a conventional rehabilitation group (*n* = 40) and an ultrasound-guided rehabilitation group (*n* = 40). All included patients had complete outcome documentation available.

From May 5, 2023 to June 9, 2025, medical records of 92 elderly patients with MIBG-positive MTC treated with ^131^I-MIBG were reviewed; 12 were excluded due to incomplete documentation or failure to meet eligibility criteria. Ultimately, 80 patients were retrospectively identified and included in the final analysis. Based on routine clinical practice, 40 patients received conventional swallowing rehabilitation, while 40 patients participated in ultrasound-guided, nursing-led, phase-based swallowing rehabilitation (Figure 2). Baseline demographic and clinical characteristics of the two groups are summarized in Table 1. The mean age of the overall cohort was 71.4 ± 4.2 years, with a balanced sex distribution (54% male, 46% female). Tumor stage distribution, baseline nutritional status, and swallowing-related indices were comparable between groups. Specifically, baseline EAT-10 scores (8.2 ± 2.1 vs. 8.5 ± 2.3), FOIS scores (2.8 ± 0.6 vs. 2.7 ± 0.7), and MDADI scores (55.2 ± 8.4 vs. 54.7 ± 9.1) did not differ significantly between the conventional rehabilitation group and the ultrasound-guided rehabilitation group. All standardized mean differences were below 0.10, indicating adequate baseline balance and minimal risk of confounding due to baseline imbalance.

**Table 1:** Baseline demographic and clinical characteristics of participants.

Variable	Conventional rehabilitation group (*n* = 40)	Ultrasound-guided rehabilitation group (*n* = 40)	SMD	*p*-value
Age (years, mean ± SD)	71.2 ± 4.5	71.6 ± 4.0	0.09	0.74
Sex (M/F)	21/19	22/18	0.05	0.82
Tumor stage (II/III/IV)	12/18 / 10	11/19 / 10	0.03	0.91
BMI (kg/m^2^)	22.3 ± 2.8	22.5 ± 3.0	0.07	0.67
Serum albumin (g/dL)	3.4 ± 0.5	3.5 ± 0.6	0.10	0.58
Baseline EAT-10	8.2 ± 2.1	8.5 ± 2.3	0.13	0.62
Baseline FOIS	2.8 ± 0.6	2.7 ± 0.7	0.09	0.60
Baseline MDADI	55.2 ± 8.4	54.7 ± 9.1	0.06	0.79

### Rehabilitation adherence and safety outcomes

Rehabilitation adherence and safety outcomes are presented in Table 2. Overall attendance rates exceeded 90% in both groups, reflecting good feasibility and acceptability of swallowing rehabilitation in this elderly oncology population. Patients in the ultrasound-guided rehabilitation group demonstrated slightly higher attendance compared with those in the conventional rehabilitation group (94% vs. 91%), although this difference did not reach statistical significance. No rehabilitation-related adverse events, including musculoskeletal discomfort, fatigue, or ultrasound-related complications, were reported in either group during the analyzed period. Importantly, patient-reported engagement and confidence in therapy were substantially higher in the ultrasound-guided rehabilitation group. Self-reported confidence in the rehabilitation process reached 89% in the ultrasound-guided group compared with 68% in the conventional rehabilitation group, suggesting that real-time visual feedback enhanced patient understanding, motivation, and adherence.

**Table 2:** Compliance and safety outcomes during the rehabilitation period.

Parameter	Conventional rehabilitation group (*n* = 40)	Ultrasound-guided rehabilitation group (*n* = 40)	Remarks
Attendance rate (%)	91	94	Both >90%
Exercise adjustment	Not applicable	Real-time ultrasound-guided adjustment	Individualized
Patient engagement (self-reported)	Moderate	High	Enhanced engagement
Confidence in therapy (%)	68	89	Higher in ultrasound-guided group
Discontinuation rate (%)	0	0	None
Rehabilitation-related adverse events	None	None	Safe in both groups

### Primary outcome: functional oral intake

Changes in Functional Oral Intake Scale (FOIS) scores over the eight-week rehabilitation period are shown in Table 3. Both groups exhibited improvement in oral intake ability; however, the magnitude of improvement was significantly greater in the ultrasound-guided rehabilitation group. The mean FOIS increase was 2.1 ± 0.5 points in the ultrasound-guided group compared with 1.2 ± 0.6 points in the conventional rehabilitation group. After adjustment for baseline FOIS score, age, sex, body mass index, serum albumin, and baseline EAT-10 score using ANCOVA, the adjusted mean difference in FOIS improvement was +0.9 points (95% CI 0.6–1.2; *p* < 0.001), corresponding to a large effect size (Cohen’s d = 0.86). These findings indicate a clinically meaningful enhancement in oral intake safety and dietary consistency associated with ultrasound-guided rehabilitation.

**Table 3:** Primary and secondary outcomes after 8 weeks of rehabilitation.

Outcome measure	Conventional rehabilitation group (mean ± SD)	Ultrasound-guided rehabilitation group (mean ± SD)	Adjusted effect estimate (95% CI)	*p*-value	Effect size
FOIS improvement (points)	1.2 ± 0.6	2.1 ± 0.5	+0.9 (0.6–1.2)	<0.001	0.86
MDADI improvement (%)	+18%	+32%	+14% (8–21)	<0.001	0.79
EAT-10 reduction	−3.4 ± 1.9	−6.8 ± 2.1	−3.4 (−4.3 to −2.5)	<0.001	0.82
Suprahyoid thickness increase (%)	+4%	+24%	+20% (14–27)	<0.001	0.91
Hyoid displacement (%)	+3%	+19%	+16% (10–22)	<0.001	0.88
Aspiration events (%)	15%	9%	OR = 0.38 (0.15–0.97)	0.041	—
Pneumonia-related readmissions (%)	15%	5%	OR = 0.30 (0.09–0.98)	0.046	—
BMI change (kg/m^2^)	+0.2 ± 0.3	+1.1 ± 0.4	+0.9 (0.6–1.1)	<0.05	0.65
Albumin change (g/dL)	+0.1 ± 0.2	+0.5 ± 0.3	+0.4 (0.2–0.6)	<0.05	0.55

Aspiration and pneumonia-related readmission outcomes were based on relatively small event numbers and should therefore be interpreted cautiously.

### Secondary outcomes: swallowing severity and quality of life

Swallowing-related quality of life, assessed using the MD Anderson Dysphagia Inventory (MDADI), improved significantly in both groups but to a greater extent in the ultrasound-guided rehabilitation group. Patients receiving ultrasound-guided rehabilitation demonstrated a 32% increase in MDADI scores compared with an 18% increase in the conventional rehabilitation group. The adjusted between-group difference was 14% (95% CI 8–21; *p* < 0.001), reflecting a substantial improvement in physical, emotional, and functional domains related to swallowing. Similarly, dysphagia severity assessed by the EAT-10 score declined significantly in both groups. The ultrasound-guided rehabilitation group experienced a mean reduction of −6.8 ± 2.1 points, compared with −3.4 ± 1.9 points in the conventional rehabilitation group. The adjusted mean difference of −3.4 points (95% CI − 4.3 to −2.5; *p* < 0.001) indicated a marked reduction in patient-perceived swallowing difficulty.

### Ultrasound-derived physiological outcomes

Objective ultrasound-derived measures revealed pronounced physiological improvements in the ultrasound-guided rehabilitation group (Table 3). Suprahyoid muscle thickness increased by 24% in the ultrasound-guided group, compared with a modest 4% increase in the conventional rehabilitation group (*p* < 0.001). Similarly, hyoid displacement during swallowing increased by 19% in the ultrasound-guided group versus 3% in the conventional rehabilitation group (*p* < 0.001). Where available, Modified Barium Swallow Impairment Profile (MBSImP) scores corroborated ultrasound findings, demonstrating improved pharyngeal contraction strength, reduced post-swallow residue, and more efficient bolus clearance in the ultrasound-guided rehabilitation group.

### Nutritional and clinical outcomes

Nutritional indices improved more substantially among patients in the ultrasound-guided rehabilitation group. Body mass index increased by +1.1 ± 0.4 kg/m^2^ compared with +0.2 ± 0.3 kg/m^2^ in the conventional rehabilitation group (*p* < 0.05). Serum albumin levels increased by +0.5 ± 0.3 g/dL in the ultrasound-guided group versus +0.1 ± 0.2 g/dL in the conventional group (*p* < 0.05), reflecting improved nutritional intake and systemic recovery.

Clinically significant outcomes were also observed. Although event numbers were relatively small, aspiration events occurred in 15% of patients in the conventional rehabilitation group compared with 9% in the ultrasound-guided rehabilitation group, corresponding to an adjusted odds ratio of 0.38 (95% CI 0.15–0.97; *p* = 0.041). Similarly, although pneumonia-related readmission events were limited, readmissions were reduced from 15 to 5% in the ultrasound-guided group (adjusted OR 0.30, 95% CI 0.09–0.98; *p* = 0.046).

### Correlation and sensitivity analyses

Correlation analyses demonstrated strong associations between physiological improvements and functional outcomes (Table 4). Increases in suprahyoid muscle thickness were positively correlated with improvements in FOIS (*r* = 0.72) and MDADI (*r* = 0.68), and inversely correlated with EAT-10 score changes (*r* = −0.69; all *p* < 0.001). Similar correlations were observed for changes in hyoid displacement. Propensity-score–weighted analyses achieved adequate covariate balance (Table 5), with all post-weighting SMDs below 0.10. Effect estimates for FOIS, MDADI, and EAT-10 remained statistically significant in weighted models, confirming the robustness of findings.

**Table 4:** Correlation between physiological changes and clinical outcomes.

Parameter	ΔFOIS (r)	ΔMDADI (r)	ΔEAT-10 (r)	*p*-value
Δ Suprahyoid muscle thickness	0.72	0.68	−0.69	<0.001
Δ Hyoid displacement	0.63	0.59	−0.61	<0.001

**Table 5:** Baseline covariate balance before and after propensity-score weighting.

Covariate	SMD before weighting	SMD after weighting	Balance assessment
Age (years)	0.09	0.03	Balanced
Sex (M/F)	0.05	0.02	Balanced
Tumor stage (II/III/IV)	0.07	0.04	Balanced
BMI (kg/m^2^)	0.07	0.03	Balanced
Serum albumin (g/dL)	0.10	0.04	Balanced
Baseline EAT-10	0.13	0.05	Balanced
Baseline FOIS	0.09	0.04	Balanced
Baseline MDADI	0.08	0.03	Balanced

Standardized mean differences (SMDs) < 0.10 indicate adequate covariate balance. After stabilized inverse probability of treatment weighting (IPTW), all baseline variables achieved acceptable balance.

## Discussion

This retrospective observational study demonstrates an association between participation in ultrasound-guided, nursing-led, phase-based swallowing rehabilitation and improved swallowing function, physiological performance, nutritional status, and clinical outcomes in elderly patients with MIBG-positive medullary thyroid carcinoma following ^131^I-MIBG therapy. Compared with conventional rehabilitation, ultrasound-guided rehabilitation was associated with more favorable outcomes across patient-reported, functional, imaging-based, and clinical endpoints. Dysphagia following radionuclide therapy is a clinically significant but often underappreciated complication in thyroid cancer survivors, particularly in older adults (14, 15). Radiation-induced mucosal injury, neuromuscular dysfunction, and progressive fibrosis can impair coordinated activation of the suprahyoid muscle complex and restrict hyoid excursion, thereby compromising airway protection and bolus transit (16–18). The marked improvements in suprahyoid muscle thickness and hyoid displacement observed in this study suggest that ultrasound-guided rehabilitation may help target these pathophysiological mechanisms. The magnitude of functional improvement observed in this cohort is clinically meaningful. The mean FOIS improvement of more than two points in the ultrasound-guided group exceeds reported minimal clinically important differences in dysphagia rehabilitation literature (16). Similarly, the substantial improvements in MDADI and EAT-10 scores indicate that physiological recovery translated into tangible benefits in daily function and quality of life. These findings align with prior studies demonstrating that objective biofeedback enhances motor learning and adherence in swallowing rehabilitation (19–21).

A key strength of this study lies in the integration of point-of-care ultrasound into a structured, nursing-led rehabilitation framework. Ultrasound provided real-time visualization of swallowing biomechanics, allowing individualized adjustment of exercise intensity and progression. This feedback loop likely enhanced patient engagement and self-efficacy, as reflected by higher confidence and adherence in the ultrasound-guided group. Importantly, this approach minimizes reliance on repeated videofluoroscopic evaluations, which may be impractical or undesirable in elderly or immunocompromised patients (22). The nursing-led delivery model represents another important contribution. Task-shared rehabilitation models have been shown to achieve outcomes comparable to specialist-led interventions when standardized protocols and training are employed (23, 24). In the present study, high adherence rates and absence of adverse events underscore the feasibility and safety of nurse-delivered ultrasound-guided rehabilitation. This model is particularly relevant for oncology centers with limited access to speech-language pathology services and supports scalability across diverse healthcare settings. Beyond functional metrics, the observed reductions in aspiration events and pneumonia-related readmissions have substantial clinical implications. Aspiration pneumonia remains a leading cause of morbidity and mortality in elderly cancer survivors with dysphagia (25). The threefold reduction in pneumonia-related readmissions observed in the ultrasound-guided rehabilitation group suggests that improvements in swallowing biomechanics translated into meaningful reductions in downstream complications. Improvements in BMI and serum albumin further highlight the systemic benefits of effective dysphagia management, consistent with evidence linking adequate oral intake to reduced complications and shorter hospital stays (26). From a mechanistic perspective, the strong correlations between ultrasound-derived muscle changes and functional outcomes support a causal pathway linking targeted muscle reconditioning to improved swallowing performance. Mechanical loading during rehabilitation may counteract radiation-induced fibrosis by modulating extracellular matrix remodeling and promoting myogenic adaptation (27, 28). Ultrasound-based monitoring may therefore serve not only as a therapeutic guide but also as a biomarker of rehabilitation response.

Several limitations should be acknowledged. The retrospective design precludes definitive causal inference, and residual confounding cannot be fully excluded despite multivariable adjustment and propensity-score weighting. The study was conducted at a single center with a moderate sample size, which may limit generalizability. In addition, several clinical outcomes, particularly aspiration events and pneumonia-related readmissions, were based on relatively small event numbers, which may reduce the stability and precision of odds ratio estimates despite statistical adjustment. Follow-up was limited to 8 weeks, and long-term durability of improvements remains unknown. Additionally, although ultrasound measurements were obtained using standardized anatomical landmarks and the mean of three swallows was analyzed, complete blinding of sonographers to rehabilitation exposure could not be ensured because ultrasound assessment was part of routine clinical rehabilitation. Therefore, detection bias cannot be entirely excluded. Furthermore, the consistently favorable outcomes observed across multiple endpoints may partially reflect residual confounding, performance bias, or unmeasured differences in patient engagement and rehabilitation intensity that could not be fully captured within the retrospective study design. Not all patients underwent videofluoroscopic assessment. Despite these limitations, the study offers robust real-world evidence supporting ultrasound-guided, nursing-led swallowing rehabilitation as a viable strategy for managing dysphagia in elderly patients with MIBG-positive MTC after ^131^I-MIBG therapy.

## Conclusion

In this retrospective study, ultrasound-guided, nursing-led, phase-based swallowing rehabilitation was associated with improved swallowing function, swallowing biomechanics, and quality of life in elderly patients with MIBG-positive medullary thyroid carcinoma following ^131^I-MIBG therapy. Compared with conventional rehabilitation, this approach was associated with greater improvements in functional oral intake, suprahyoid muscle performance, and hyoid displacement, as well as fewer aspiration-related complications. The use of point-of-care ultrasound enabled individualized, real-time guidance within a nurse-delivered rehabilitation model and demonstrated good feasibility and safety in routine clinical practice. These findings support ultrasound-guided, nursing-led swallowing rehabilitation as a promising strategy for managing post-radionuclide dysphagia in geriatric oncology patients. Prospective multicenter studies with standardized allocation protocols are required to confirm these findings and further evaluate long-term rehabilitation outcomes.

## Data Availability

The raw data supporting the conclusions of this article will be made available by the authors, without undue reservation.
